# Editorial: Community series in epigenetics in the microbiome-host crosstalk: from mechanisms to therapeutics, volume II

**DOI:** 10.3389/fimmu.2026.1929447

**Published:** 2026-07-28

**Authors:** Hao-Yu Liu, Chengfei Liu, Omar Ramos-Lopez

**Affiliations:** 1 College of Animal Science and Technology, Yangzhou University, Yangzhou, China; 2 Joint International Research Laboratory of Agricultural and Agri-Product Safety, The Ministry of Education of China, Yangzhou University, Yangzhou, China; 3 Department of Urologic Surgery, University of California, Davis, Davis, CA, United States; 4 Davis Comprehensive Cancer Center, University of California, Sacramento, CA, United States; 5 Medicine and Psychology School, Autonomous University of Baja California, Tijuana, Mexico

**Keywords:** bacteria, dysbiosis, epigenetic, microbiome, microbiota, therapeutics

The collection of microorganisms that colonize the human gastrointestinal tract is now recognized as a key regulator of host physiology, affecting processes that range from metabolism and intestinal barrier function to the development of the immune system. One major route through which the microbiota exerts these effects is the induction of epigenetic changes, *i.e.*, heritable shifts in gene expression that operate without altering the genomic DNA sequence. Mechanistically, microbial metabolites including short-chain fatty acids, methyl donor compounds (*e.g.*, S-adenosylmethionine), and tryptophan-derived ligands can serve as enzymatic cofactors or modify the activity of writers and erasers of DNA and histone marks. In parallel, microbiota-triggered signaling cascades intersect with chromatin-remodeling complexes and non-coding RNA networks (1). When these host-microbe epigenetic circuits become imbalanced, they can contribute to the development of chronic diseases, ranging from metabolic syndrome and autoimmunity to neurological disorders and some types of cancer (2, 3). This second volume of the Community Series brings together six articles that further dissect the molecular basis of microbiota-epigenome crosstalk and explore how this knowledge can be translated into novel diagnostic and therapeutic approaches.

To explore the role of epigenetic modifications in tumor metabolism and autoimmunity, Lv et al. reviewed the emerging role of histone lactylation as a cross-regulatory node that links cellular metabolism to epigenetic reprogramming. They discussed how lactate, which can originate from both tumor cells and the microbiota, drives gene expression changes that promote immune evasion and drug resistance, and they highlighted novel strategies for targeting lactylation writers and erasers in cancer therapy. In the context of immune-mediated disorders, Mu et al. provided a comprehensive view of epigenetic dysregulation in autoimmune diseases. They described how DNA methylation, histone modifications, and non-coding RNAs contribute to the breakdown of self-tolerance under the influence of microbial triggers, emphasizing the potential of epigenetic editing to restore immune homeostasis.

Extending our understanding beyond, He et al. discovered that *Bacteroides ovatus*-derived N-methylserotonin inhibits CRC progression by binding to the serotonin receptor HTR1D and activating the cAMP-PKA pathway, which subsequently suppresses NF-κB signaling. This metabolite-receptor axis identifies a direct mechanism by which a specific gut microbe can influence host cell fate beyond the gut. Complementing this metabolite-focused work, Liu and Qi systematically reviewed the role of intratumoral microbiota in CRC pathogenesis. The authors discussed the carcinogenic activities of candidate onco-microbes such as *Fusobacterium nucleatum*, enterotoxigenic *Bacteroides fragilis*, and colibactin-producing *Escherichia coli*, focusing on mechanisms including chronic inflammation, genotoxic DNA damage, immune evasion, metabolic reprogramming, and non-coding RNA regulation. They further evaluated the diagnostic and prognostic potential of intratumoral microbial signatures and assessed emerging microbiome-targeted strategies-bacteriophages, phage-guided nanocarriers, and fecal microbiota transplantation for CRC therapy.

Extending the view beyond composition to community organization, Ni et al. introduced a social-architecture perspective on gut microbiota dynamics. By considering the microbiota as a spatially structured society with cooperative and competitive interactions, they argued that properties such as spatial segregation and metabolic cross-feeding influence host epigenetic landscapes and disease susceptibility, offering a conceptual framework for future host-microbiota studies.

The microbiota-gut-brain axis, a bidirectional connection between the gut microbiota and the brain, may also be disrupted by external factors with consequences for neurological function. In this regard, Zou et al. investigated the effects of antibiotic-induced dysbiosis on seizure susceptibility. Using ciprofloxacin treatment in a mouse model, they found that perturbed gut microbiota increased seizure susceptibility through altered hypothalamic-pituitary-adrenal axis activity and elevated inflammatory cytokines. These findings suggest that microbiota-driven neuroinflammation, likely involving epigenetic reprogramming of central immune cells, can prime the brain for hyperexcitability and highlight the neurological risks associated with antibiotic use.

Together, the contributions to this volume highlight the multifaceted interplay between microbial metabolites and host chromatin, from direct histone modification by bacterially derived small molecules to the broader influence of community-level organization on gene regulation. These studies illustrate how epigenetic mechanisms serve as a common thread linking gut dysbiosis to immune dysfunction, tumor biology, and neuroinflammation, and they point to the therapeutic potential of intercepting these pathways. Continued progress will rely on holistically integrating omics sciences, such as metabolomic and epigenomic profiling, with well-defined clinical cohorts, and on accounting for lifestyle and environmental factors (exposome) that shape both the microbiota and the host epigenome.
